# Gender Differences in Caregiver Burden, Social Support, and Coping Strategies Among Caregivers of Patients With Epilepsy in a Tribal Region of India: A Cross-Sectional Study

**DOI:** 10.7759/cureus.89695

**Published:** 2025-08-09

**Authors:** Nazish Fatima, Abid Rizvi

**Affiliations:** 1 Psychiatric Social Work, William R Sharpe Jr Hospital, Weston, USA; 2 Psychiatry and Behavioral Sciences, West Virginia University, Morgantown, USA

**Keywords:** caregiver burden, coping, epilepsy, seizure, social support

## Abstract

This study examined whether caregivers of male or female patients with epilepsy differ in perceived burden, social support, and coping mechanisms. In a cross-sectional design conducted at a tertiary neuropsychiatric hospital, 60 caregivers (30 per group) completed the Family Burden Interview Schedule (FBIS), the Social Support Questionnaire (SSQ), and the Ways of Coping Questionnaire (WCQ). Caregivers of female patients reported greater financial strain and more pronounced impacts on mental and physical health, yet they also perceived higher levels of social support. In contrast, caregivers of male patients employed more active coping strategies, such as confronting and self-controlling coping, but reported lower perceived support. Among caregivers of male patients, higher levels of social support were associated with lower perceived burdens; this relationship was not observed among caregivers of female patients. The findings underscore the importance of gender-sensitive approaches in reducing caregiver burden and enhancing care outcomes in epilepsy management.

## Introduction

Epilepsy is one of the most prevalent chronic neurological diseases, affecting 50 million people worldwide, nearly 80% of whom live in low- and middle-income countries. The latest World Health Organization estimates that up to 70% of cases could be rendered seizure-free with appropriate care. Yet, three-quarters of those in low-income settings remain untreated, intensifying reliance on family caregivers and magnifying the psychosocial toll they face [1].

There is a high objective and subjective burden, poorer physical and mental health, disrupted family functioning, and significant financial strain among epilepsy caregivers in India [2]. Furthermore, disability severity and rural residence independently predicted a heavier burden among caregivers of adults with epilepsy who also had physical impairments [3]. Other studies have found significant anxiety or depression in over half of parents caring for children with epilepsy [4]. Comparable findings have been reported in neighboring South-Asian settings, where poorly controlled seizures and early age-of-onset were linked to mood symptoms in caregivers [5].

Across this body of work, social support repeatedly emerges as a key protective factor. A 2025 mixed-methods study showed that stronger perceived support predicted lower caregiver distress and more active, problem-focused coping among parents of children with neuro-developmental disorders [6]. Interestingly, Indian literature on mental health caregiving consistently demonstrates heavier domestic and emotional loads on women, while a 2023 comparative study of schizophrenia caregivers found that differences in coping style, rather than absolute burden, distinguished male and female carers [7,8]. A recent critical review of adult-epilepsy caregivers likewise found patient gender as a potential moderator of caregiver stress, urging culturally nuanced analyses [9].

Nonetheless, a significant gap persists in understanding how patients' gender may influence caregiver experiences, particularly within tribal populations. Cultural beliefs, gender norms, societal expectations, and stigmatization associated with chronic illnesses likely differentially affect caregiver burden, social support accessibility, and coping mechanisms contingent upon the patient's gender. Exploring these gender-specific differences is crucial to developing targeted interventions that effectively address caregivers' distinct needs and enhance their caregiving experiences.

This study aimed to compare perceived caregiver burden, social support, and coping strategies among caregivers of male versus female epilepsy patients. We hypothesize that caregivers of female patients would experience higher burdens due to societal expectations, although they might also perceive stronger social support from family and community networks.

## Materials and methods

Study design and participants

This study adhered to the Strengthening the Reporting of Observational Studies in Epidemiology (STROBE) guidelines and relevant recommendations from the EQUATOR network [10]. Participants were enrolled from the outpatient department of a tertiary neuropsychiatric hospital between April 2014 and March 2016.

The study included patients between the ages of 18 and 45 years who had a confirmed diagnosis of epilepsy based on the ICD-10 Diagnostic Criteria for Research [11]. Eligible participants were required to have had the illness for a duration of at least two years and to possess a minimum of primary-level education. Patients were excluded if they had significant physical or psychiatric comorbidities or an intellectual disability.

Caregivers eligible for inclusion were immediate family members, such as a parent, spouse, sibling, or adult child, who had been living with the patient for a minimum of two years. Caregivers also had to be 18 years or older, free from any significant physical, psychiatric, or neurological disorders, and have completed at least a primary-level education.

A 2012 Indian study of family caregivers for adults with chronic mental illness found that caregivers of female patients scored, on average, six points higher on the Family Burden Interview Schedule (FBIS) than those of male patients (SD≈8; Cohen’s d≈0.70) [7]. Treating this as the smallest clinically important gap, we used G*Power 3.1 (Heinrich-Heine-Universität Düsseldorf, Düsseldorf, Germany) to compute the sample needed for an independent-samples t-test (two-tailed, α=0.05, 1-β=0.80). An effect size of 0.70 requires 28 caregiver-patient dyads per group (56 total). Anticipating up to 30% exclusions or incomplete questionnaires, we planned to recruit 56÷0.70 ≈ 80 dyads and set a round target of 90. We ultimately screened 88 dyads and retained complete data from 60 (30 female patients, 30 male patients), comfortably exceeding the minimum to maintain 80% power.

Consent

Written informed consent was obtained from all participants. Initially, 88 caregiver-patient pairs were screened; 60 pairs met the inclusion criteria and completed the assessments, with an equal number in each patient gender group (30 pairs per gender group).

Ethical Considerations

This study was conducted in accordance with the ethical principles outlined in the Declaration of Helsinki and adhered to the national ethical guidelines for biomedical and health research involving human participants [12]. Prior to the initiation of the study, the research protocol, informed consent forms, and all relevant documents were reviewed and approved by the Institutional Ethics Committee of the Ranchi Institute of Neuropsychiatry and Allied Sciences (RINPAS), Ranchi, Jharkhand, India. The date of approval was March 12, 2014.

Written informed consent was obtained from all participants after providing a clear explanation of the nature, objectives, procedures, potential risks, and benefits of the study in a language understandable to them. For participants who were unable to provide informed consent due to cognitive or psychiatric impairment, consent was obtained from a legally authorized representative in accordance with ethical and legal requirements.

All data were anonymized to maintain participant confidentiality. Access to identifiable information was restricted to authorized personnel only. Participation in the study was voluntary, and participants were assured that they could withdraw at any time without affecting their clinical care or legal standing.

The investigators affirm that all procedures involving human participants were carried out with due consideration to their dignity, rights, safety, and well-being. The study posed no foreseeable risk beyond routine clinical or psychological assessment, and appropriate measures were in place to manage any distress or adverse events arising during the research process.

Measures

The Socio-Demographic and Clinical Data Sheet was a structured checklist that recorded each patient’s age, sex, education, seizure type, current anti-seizure medicines, and the caregiver’s relationship to the patient, co-residence status, and total years of hands-on care. Because the form simply captures factual characteristics, issues of scale reliability or validity do not apply.

The FBIS, created by Pai and Kapur, contains 24 items that rate six objective burden domains-financial cost, disruption of household routines, loss of leisure, strain on family interactions, and effects on the caregiver’s physical and mental health-on a 0 (“none”) to 2 (“severe”) scale [13]. A single four-point item gauges the caregiver’s subjective sense of burden. In the original Indian field study, the objective index showed excellent internal consistency (Cronbach’s α=0.86) and four-week test-retest reliability of 0.83. 

The Social Support Questionnaire (SSQ-18, Indian adaptation), developed by Nehra and Kulhara, asks caregivers to rate 18 statements across emotional, practical, and informational support on a four-point “never” to “always” scale (total score 18-72; higher=stronger perceived support) [14]. Psychometric evaluation in family-caregiving samples produced a Cronbach’s α of 0.83 and a two-week test-retest correlation of 0.78 [14]. Exploratory factor analysis replicated the intended three-factor structure, which together explained 56% of the variance [14] (Appendix of Table 7).

The Ways of Coping Questionnaire (WCQ-66) by Folkman and Lazarus measures the frequency with which eight coping strategies are used, rating each of 66 items from 0 (“not used”) to 3 (“used a great deal”) [15]. Reliabilities for the eight sub-scales in the original manual ranged from α=0.61 to 0.79, and 12-week test-retest coefficients ranged from 0.60 to 0.75 [10]. Independent validation with Indian caregivers replicated the eight-factor solution and supported convergent and discriminant validity vis-à-vis burden and social support measures [15] (Appendix of Table 7).

All three copyrighted instruments (FBIS, SSQ-18, and WCQ-66) were sourced through our institutional library, which holds licences permitting non-commercial reproduction, administration, and publication in academic research.

Statistical Analysis

All analyses were performed in IBM SPSS Statistics 22.0. Descriptive statistics (means±SD for continuous variables; frequencies and percentages for categorical variables) were first calculated for the entire sample and separately for caregivers of male and female patients. Group differences on continuous outcomes (FBIS, SSQ, and WCQ scores) were tested with independent-samples t-tests. Socio-demographic variables were compared with Pearson’s χ² test (or Fisher’s exact test when expected cell counts were <5). Finally, Pearson’s correlation coefficients were computed to examine the relationships among caregiver burden, perceived social support, and coping strategies. Statistical significance for all procedures was set at two-tailed p<0.05.

## Results

The overall sample comprised 60 caregiver-patient dyads, split evenly by patient gender.

The two caregiver groups were broadly comparable in terms of demographics (Table 1). Mean caregiver age was slightly higher in the female patient group and reached statistical significance (t=2.13, P=0.04). In contrast, no group differences emerged for patient age, education, family structure, or household income (all P>0.05).

**Table 1:** Selected socio‑demographic characteristics of caregivers by patient gender ^*^indicates P<0.05. SD: standard deviation

Variable	Male patient caregivers (n=30)	Female patient caregivers (n=30)	χ²/*t*	P
Caregiver age, mean±SD (y)	29.87±6.90	33.77±7.30	2.13	0.04*
Patient age, mean±SD (y)	27.60±4.58	25.66±4.57	1.64	0.11
Education ≥primary, n (%)	30 (100)	30 (100)	-	-
Family with income	12 (40)	16 (53)	1.23	0.27
Nuclear family, n (%)	16 (53)	12 (40)	1.07	0.30

As summarized in Table 2, caregivers of female patients reported materially heavier burdens in several FBIS domains. Financial strain was greater (t=2.31, P=0.02). Marked group differences also appeared for effects on caregivers’ physical health (t=3.23, P=0.002) and mental health (t=4.32, P<0.001). Subjective burden scores echoed these patterns, averaging 42% higher in the female patient group (t=4.56, P<0.001). Other FBIS domains did not differ significantly between groups.

**Table 2:** Comparison of FBIS scores *indicates P<0.05. The authors obtained appropriate permission to use the FBIS for the purposes of this study. FBIS: Family Burden Interview Schedule

FBIS domain	Male patient caregivers, mean±SD	Female patient caregivers, mean±SD	t	P
Financial burden	3.90±2.12	5.13±2.00	2.31	0.02^*^
Disruption of routine activities	2.20±1.80	2.40±1.90	0.47	0.64
Disruption of family leisure	1.90±1.42	1.73±1.04	-0.46	0.65
Disruption of family interactions	1.67±0.88	1.33±0.88	-1.51	0.14
Effect on physical health	1.53±0.90	2.50±1.38	3.23	0.002^*^
Effect on mental health	1.97±0.93	2.93±0.78	4.32	<0.001^*^
Objective burden (total)	13.44±3.77	12.80±3.53	-0.67	0.51
Subjective burden	1.60±0.49	2.27±0.64	4.56	<0.001^*^

Consistent with the heavier objective and subjective loads, caregivers of female patients simultaneously perceived substantially stronger social support (Table 3), exceeding that of the male patient group by one standard deviation (t=3.81, P<0.001).

**Table 3:** Perceived social support *indicates P<0.05. The authors obtained appropriate permission to use the SSQ for the purposes of this study.

Variable	Male patient caregivers, mean±SD	Female patient caregivers, mean±SD	t	P
Social support (total)	31.33±9.35	41.77±11.68	3.81	<0.001*

By contrast, coping profiles favored caregivers of male patients (Table 4). They employed confrontive actions, self‑control, deliberate problem solving, positive reappraisal, and escape‑avoidance significantly more often than their counterparts (all P≤0.02). These skills aggregated into a markedly higher total WCQ score (t=6.65, P<0.001).

**Table 4:** WCQ *indicates P<0.05. The authors obtained appropriate permission to use the WCQ for the purposes of this study. WCQ: Ways of Coping Questionnaire

WCQ domain	Male patient caregivers, mean±SD	Female patient caregivers, mean±SD	t	P
Confrontive coping	9.13±2.50	6.44±1.90	4.68	<0.001*
Distancing	11.00±2.10	9.50±2.30	2.34	0.02*
Self‑controlling	14.32±2.50	10.11±2.70	5.90	<0.001*
Seeking social support	11.22±3.00	7.80±1.90	5.17	<0.001*
Accepting responsibility	7.30±1.90	6.10±1.50	2.99	0.004*
Escape–avoidance	15.74±3.61	9.60±2.80	7.37	<0.001*
Planful problem solving	11.23±3.70	9.12±3.00	2.49	0.02*
Positive reappraisal	12.80±3.23	8.23±3.20	5.50	<0.001*
Total coping score	76.65±9.60	62.77±6.60	6.65	<0.001*

Correlation analysis revealed a moderate, inverse association between perceived social support and caregiver burden within the male patient group (r=-0.43, P=0.02; Table 5).

**Table 5:** Correlations among caregivers of male patients (n=30) *indicates P<0.05.

Variable	1 (burden)	2 (social support)	3 (coping)
1. Burden	1	-	-
2. Social support	-0.43*	1	-
3. Ways of coping	0.14	-0.08	1

No comparable associations emerged in the female patient group (Table 6), suggesting that the buffering influence of support may operate differently across gendered caregiving contexts.

**Table 6:** Correlations among caregivers of female patients (n=30)

Variable	1 (burden)	2 (social support)	3 (coping)
1. Burden	1	-	-
2. Social support	-0.34	1	-
3. Ways of coping	-0.11	0.15	1

## Discussion

The present study adds weight to previous research showing that families caring for women with chronic neurological disorders often face heavier impacts on caregivers’ physical and mental health, likely reflecting gendered stigma around household roles [7,16]. Conversely, caregivers of male patients reported higher scores on confrontive and problem-solving coping, a profile previously linked to maintaining masculine identities within the family. Perceived social support was higher in the female patient group and, within the male patient group only, was inversely related to burden (r=-0.43, p=0.02). Because that correlation did not reach conventional significance in the female patient group (r=-0.34, p=0.11), and we did not perform a Fisher r-to-z comparison, we cannot claim a statistically proven gender difference in the strength of this association. Instead, the pattern should be viewed as preliminary and hypothesis-generating: larger, adequately powered studies are required to verify whether social support truly moderates caregiver burden differently for male versus female patient dyads.

This study advances the small but growing body of work on gendered caregiving in chronic neurological illness in several key ways. First, by recruiting comparable samples of caregivers across patient gender within the same sociocultural milieu, we were able to tease apart dyad‑level differences from broader contextual influences. The markedly heavier FBIS financial and health‑impact scores among caregivers of female patients echo qualitative evidence from rural South Asia suggesting that daughters and wives with epilepsy are often viewed as long‑term dependents whose illness jeopardizes household economic stability and marital prospects [16]. In such settings, stigma surrounding epilepsy can be gendered, limiting educational and occupational opportunities for women and thereby amplifying the material strain borne by their families.

Paradoxically, the same cultural norms that magnify both objective and subjective burdens may simultaneously mobilize extended kin networks in support of female patients, explaining the higher social-support scores we observed. Similar "compensatory" effects have been documented in caregivers of daughters with intellectual disability, where communal expectations of shared responsibility buffer psychological distress [17]. The inverse association between support and burden among male patient caregivers, but not among female patient caregivers, further underscores that social support operates as a context-dependent resource rather than a universal antidote; its protective power appears most excellent where baseline support is relatively scarce.

Coping strategy profiles lend additional nuance. Caregivers of male patients reported greater use of confrontive, planful, and reappraisal‑based coping, aligning with prior findings that male illness often entails pressures for caregivers, frequently spouses or mothers, to "solve" problems discreetly so that masculine roles are preserved [18]. By contrast, the heavier reliance on communal support among caregivers of female patients may attenuate the need for highly active, individual coping responses.

Several limitations temper our conclusions. The cross‑sectional design precludes causal inference about the dynamics among burden, support, and coping. Self-report instruments, although well-validated, are susceptible to social-desirability bias, particularly around culturally sensitive topics such as financial hardship. Generalizability is constrained by the study’s single-center, tertiary-hospital sample and its exclusive focus on caregivers meeting the minimum literacy criteria. Nonetheless, strengths include rigorous adherence to STROBE guidelines, balanced group sizes, and the use of multidimensional measures of burden, support, and coping.

Implications for practice are clear. Interventions for caregivers of female patients should prioritize structured stress-management and financial-planning components, while programs for caregivers of male patients might focus on enhancing tangible and emotional support, for example, through peer-led support groups and community health-worker outreach. Given the differential patterns observed, mixed‑gender caregiver workshops that explicitly address gendered expectations could foster mutual learning and normalize diverse coping repertoires.

Future research should incorporate longitudinal designs to track how caregiver experiences evolve over the illness trajectory and through key life events such as marriage, childbirth, or employment changes. Mixed‑methods approaches could illuminate the lived experience behind the quantitative patterns identified here, and interventional trials are needed to test the efficacy of gender‑tailored support packages.

Several constraints temper our conclusions. (i) With only 30 caregivers per group, the study is powered to detect medium-to-large effects but may miss subtler differences, including potential disparities between correlation coefficients. (ii) We did not conduct a Fisher z test, so the apparent contrast in the support-burden correlation across genders could reflect sampling variability rather than a true interaction. (iii) The cross-sectional design precludes causal inference, and reliance on self-report introduces possible social-desirability bias. (iv) Participants were recruited from a single tertiary center during 2014-2016; social norms, service availability, and epilepsy care pathways may have changed since then, limiting generalizability. These factors underscore the need for larger, longitudinal, multi-site investigations that combine quantitative and qualitative methods to clarify gender-specific caregiving dynamics and to test targeted intervention strategies.

## Conclusions

This investigation demonstrates that caregiving for adults with epilepsy is not a monolithic experience, but one that is strongly conditioned by the patient’s gender. Caregivers of female patients endure heavier financial, physical‑health, and emotional loads, yet benefit from more robust social networks, whereas caregivers of male patients marshal a wider repertoire of active coping strategies despite perceiving less support. These asymmetries suggest that interventions cannot adopt a one‑size‑fits‑all approach: programs should prioritize skill‑building and stress management techniques for families of female patients, while simultaneously strengthening formal and informal support systems for those caring for male patients. Although limited by its modest, single‑center sample and cross‑sectional design, the study highlights actionable targets, particularly social support enhancement and coping strategy training, that can inform culturally sensitive, gender‑responsive caregiver services. Future longitudinal and multicenter research is warranted to confirm these patterns and evaluate the effectiveness of tailored interventions.

## Appendices

**Table 7:** Comparison of the SSQ and the WCQ (full items omitted due to copyright) The authors obtained appropriate permission to use the SSQ and the WCQ for the purposes of this study. SSQ: Social Support Questionnaire; WCQ: Ways of Coping Questionnaire

Field	SSQ	WCQ
Developer	Nehra and Kulhara (1987)	Folkman and Lazarus (1985)
Number of Items	18 items	66 items
Response format	4-point Likert scale (1=never to 4=always)	4-point Likert scale (0=not used to 3=used a great deal)
Domains	Emotional, practical, and informational support	8 subscales: confrontive, distancing, self-controlling, seeking social support, accepting responsibility, escape-avoidance, planful problem-solving, and positive reappraisal
Example item	Someone listens to your concerns	I made a plan of action and followed it
Scoring method	Sum of item scores (18-72)	Subscale scores only; no total score
Psychometric properties	Cronbach's alpha: 0.83; test-retest: 0.78	Alpha: 0.61-0.79 across subscales
Validation samples	Indian caregiver populations	Caregivers, students, and clinical populations
Administration time	10-15 minutes	20-30 minutes
